# ﻿On the synonymy of *Dactylopisthoides* Eskov, 1990 and *Uusitaloia* Marusik, Koponen & Danilov, 2001 (Araneae, Linyphiidae)

**DOI:** 10.3897/zookeys.1184.113255

**Published:** 2023-11-21

**Authors:** Andrei Tanasevitch, Yuri Marusik

**Affiliations:** 1 A.N. Severtsov Institute of Ecology and Evolution, Russian Academy of Sciences, Leninsky prospekt 33, Moscow 119071, Russia A.N. Severtsov Institute of Ecology and Evolution, Russian Academy of Sciences Moscow Russia; 2 Institute for Biological Problems of the North FEB RAS, Portovaya Str. 18, Magadan 685000, Russia Institute for Biological Problems of the North FEB RAS Magadan Russia; 3 Altai State University, Lenina Pr., 61, Barnaul, RF-656049, Russia Altai State University Barnaul Russia; 4 Department of Zoology & Entomology, University of the Free State, Bloemfontein 9300, South Africa University of the Free State Bloemfontein South Africa

**Keywords:** Asia, East Siberia, Erigoninae, new combination, taxonomy

## Abstract

Two erigonine genera from East Siberia are synonymized: *Uusitaloia* Marusik, Koponen & Danilov, 2001, **syn. nov.** = *Dactylopisthoides* Eskov, 1990. Two new combinations are established: *Dactylopisthoidestransbaicalicus* (Marusik, Koponen & Danilov, 2001), **comb. nov.** and *Dactylopisthoideswrangelianus* (Marusik & Koponen, 2009), **comb. nov.** both ex. *Uusitaloia*. The epigyne of *D.wrangelianus* is illustrated for the first time. A new, updated diagnosis of *Dactylopisthoides* is proposed. The copulatory organs of both sexes of *D.hyperboreus* and *D.wrangelianus* are illustrated by SEM images.

## ﻿Introduction

The erigonine genus *Uusitaloia* Marusik, Koponen & Danilov, 2001 was established for *Uusitaloiatransbaicalica* Marusik, Koponen & Danilov, 2001, a species that was described from the holotype male from the region of Lake Baikal, Buryatia, Russia (Marusik et al. 2001). The second congener, *U.wrangeliana* Marusik & Koponen, 2009, was described from Wrangel Island, Russia (Marusik and Koponen 2009). A detailed re-examination of the type materials of both *Uusitaloia* species has revealed that their conformation of the copulatory organs in both sexes is similar to that of the generotype of the monotypic genus *Dactylopisthoides* Eskov, 1990, known from the upper reaches of the Kolyma River. This paper is aimed at (1) establishing a synonymy of these genera, and (2) providing an updated diagnosis of the genus *Dactylopisthoides*.

## ﻿Material and methods

This paper is based on the collections deposited in the Zoological Museum of the Moscow State University, Moscow, Russia (**ZMMU**), and the specimens that are temporary in the Zoological Museum of the University of Turku, Finland (**ZMUT**). Specimens preserved in 70% ethanol were studied using an MBS-9 stereo microscope. Photographs were taken using a SEM JEOL JSM-5200 scanning microscope at the ZMUT. Leg chaetotaxy is presented as follows: e.g., 2.2.2.1, referring to a number of dorsal spines on tibiae I–IV. Terminology of the copulatory organ sclerites largely follows Merrett (1963) as well as the authors indicated in the abbreviations given below.

### ﻿Abbreviations

**DSA** distal suprategular apophysis sensu Hormiga (2000);

**EP** embolus proper sensu Saaristo (1971);

**MM** median membrane sensu van Helsdingen (1965), = embolic membrane sensu van Helsdingen (1986) and Hormiga (2000);

**MT** median tooth of DSA;

**PP** posterior projections of lateral walls of epigyne sensu Saaristo and Tanasevitch (1996);

**R** radix;

**RT** radical tooth;

**TmI** position of trichobothrium on metatarsus I.

## ﻿Taxonomy

### ﻿Class Arachnida Cuvier, 1812

**Order Araneae Clerck, 1757**

**Family Linyphiidae Blackwall, 1859**

**Subfamily Erigoninae Emerton, 1882**

#### ﻿ Dactylopisthoides

Taxon classificationAnimaliaAraneaeLinyphiidae

Eskov, 1990

4622DA4E-F997-5DA1-A2FB-563027541B8C

Dactylopisthoides Eskov, 1990: 4 (type species: Dactylopisthoideshyperboreus Eskov, 1990).
Uusitaloia Marusik, Koponen & Danilov, 2001: 89 (type species: Uusitaloiatransbaicalica Marusik, Koponen & Danilov, 2001), syn. nov.

##### Diagnosis.

**Figure 1.**
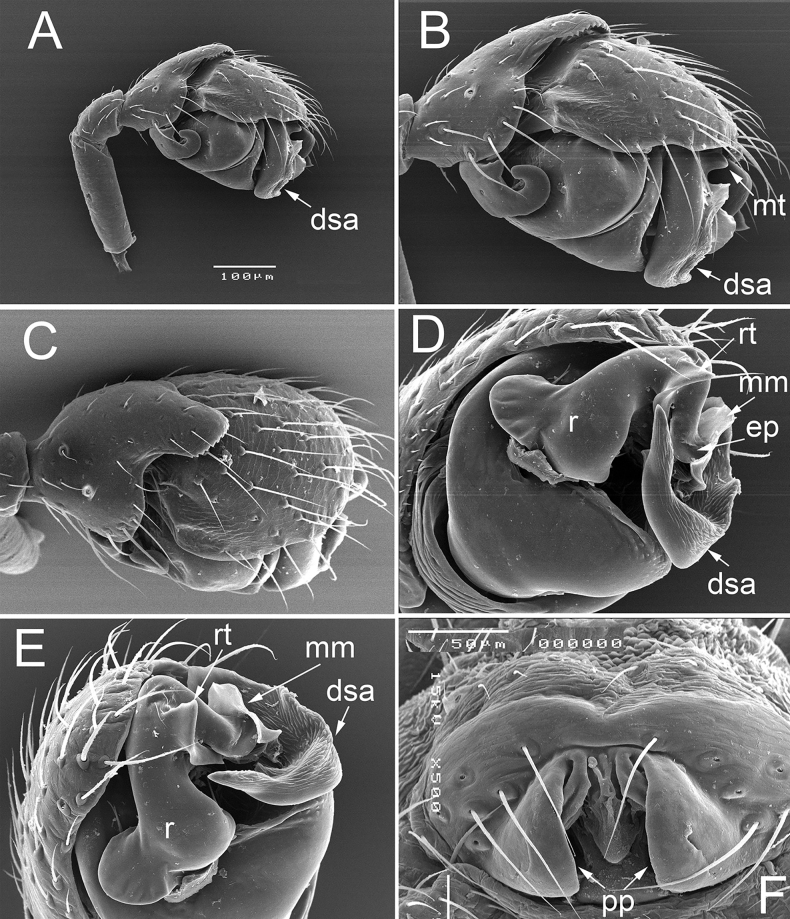
Details of the male palp (**A–E**) and epigyne (**F**) of *Dactylopisthoideshyperboreus* Eskov, 1990, specimens from the type locality **A–C** right palp, retrolateral (**A, B**) and dorsal view (**C**), respectively **D, E** bulb, lateral view, different aspects **F** epigyne, ventral view. Abbreviations: dsa – distal suprategular apophysis, ep – embolus proper, mm – median membrane, mt – median tooth, pp – posterior projections of lateral walls, r – radix, rt – radical tooth.

**Figure 2.**
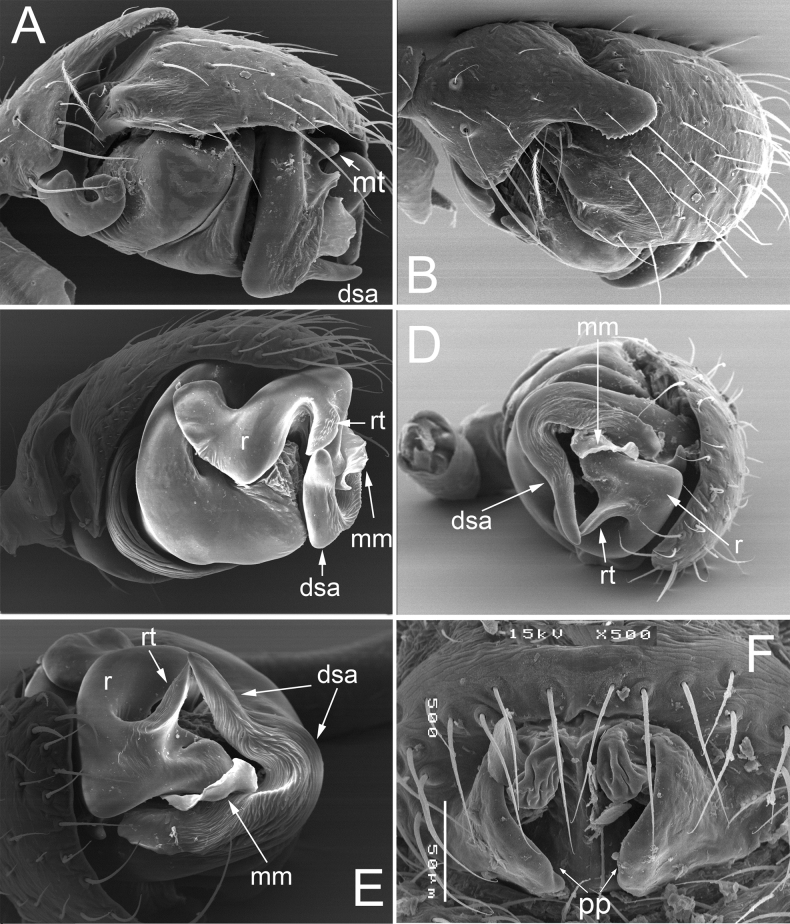
Details of the male palp (**A–E**) and female epigyne (**F**) of *Dactylopisthoideswrangelianus* (Marusik & Koponen, 2009), specimens from Mamontovaya River, Wrangel Isl **A–D** right palp, retrolateral, dorsal, ventral and frontal view, respectively **E** embolic division, frontal view **F** epigyne, ventral view. Abbreviations: dsa – distal suprategular apophysis, ep – embolus proper, mm – median membrane, mt – median tooth, pp – posterior projections of lateral walls, r – radix, rt – radical tooth.

The genus *Dactylopisthoides* is very similar to *Dactylopisthes* Simon, 1884, with the type species *D.digiticeps* (Simon, 1881). Both genera belong to the *Savignia*-genus group sensu Millidge (1977). The main diagnostic characters are as follows:

1. Formula of leg chaetotaxy: *Dactylopisthoides* – 2.2.2.1, vs 2.2.1.1 in *Dactylopisthes*;
2. Male carapace in *Dactylopisthoides* is unmodified, vs highly modified except for three Oriental species of which the generic assignment is doubtful;
3. Male palpal tibia in *Dactylopisthoides* is only slightly elongated dorsally, vs highly modified, sickle-shaped in *Dactylopisthes*, except for *D.mirabilis* (Tanasevitch, 1985), known from Kyrgyzstan (Tanasevitch 1985);
4. Epigyne in *Dactylopisthoides* with an opened fovea, its lateral walls with a distinct posterior projection on each (Figs 1F, 2F), vs fovea absent.

##### Description.

Small to medium erigonine. Male total length 1.50–1.80, carapace 0.63–0.79 long. Female total length 1.53–1.75, carapace 0.6–0.68 long. Carapace unmodified in both sexes. Formula of leg chaetotaxy 2.2.2.1, tibial spines short and weak. Metatarsi I–III each with trichobothrium. TmI 0.50–0.70. Male palpal tibia slightly modified, somewhat elongated dorsally. Paracymbium small and narrow, L-shaped, uncinate apically. Distal suprategular apophysis greatly enlarged, highly sclerotized, median tooth present. Embolic division relatively large, radix curved, with small outgrowth (= radical tooth); embolus proper short, thick, covered with short median membrane. Epigyne with cavity, lateral walls with distinct posterior projection in each.

##### Comments.

**Figure 3.**
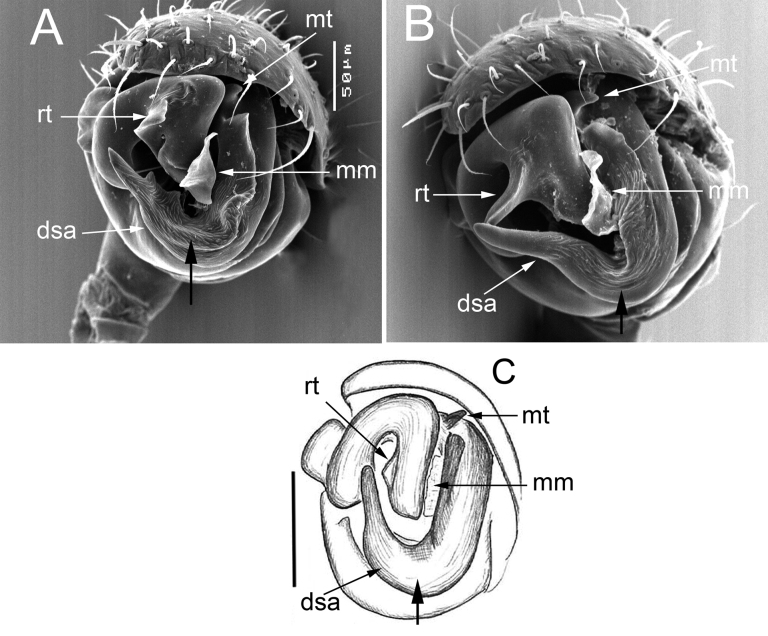
Frontal view of the embolic divisions of *Dactylopisthoideshyperboreus* Eskov, 1990 (**A**), *D.wrangelianus* (Marusik & Koponen, 2009) (**B**) and *D.transbaicalicus* (Marusik, Koponen & Danilov, 2001) [reproduced from Marusik and Koponen (2009)] (**C**). Abbreviations: dsa – distal suprategular apophysis, mm – median membrane, mt – median tooth, rt – radical tooth. Black, thick arrows show additional differences between species. Scale bar: 0.1 mm (**С**).

An analysis of both somatic and copulatory organ structures of the type species of *Uusitaloia* and *Dactylopisthoides* has revealed that they are undoubtedly congeneric: all the species in both genera are of a similar small size, possess the same chaetotaxy (2221) and trichobothriotaxy (I–III; TmI: 0.64–0.65), and are characterized by the same conformation of copulatory organs in both sexes, differing from each other only in minor details: cf. Figs 1–3. Thus, the genus *Uusitaloia* Marusik, Koponen & Danilov, 2001 is to be considered a junior synonym of *Dactylopisthoides* Eskov, 1990.

In the papers dealing with *Dactylopisthoides* (Eskov 1990) and *Uusitaloia* (Marusik et al. 2001; Marusik and Koponen 2009), the authors provided different tibial chaetotaxy even for different sexes. The reason for this appears to have been the small size of the spines, which could be easily overlooked (for further details, see Tanasevitch 2022).

##### Composition.

The genus *Dactylopisthoides* currently consists of three very similar species: *D.hyperboreus* Eskov, 1990; *D.transbaicalicus* (Marusik, Koponen & Danilov, 2001) and *D.wrangelianus* (Marusik & Koponen, 2009).

##### Distribution.

**Figure 4.**
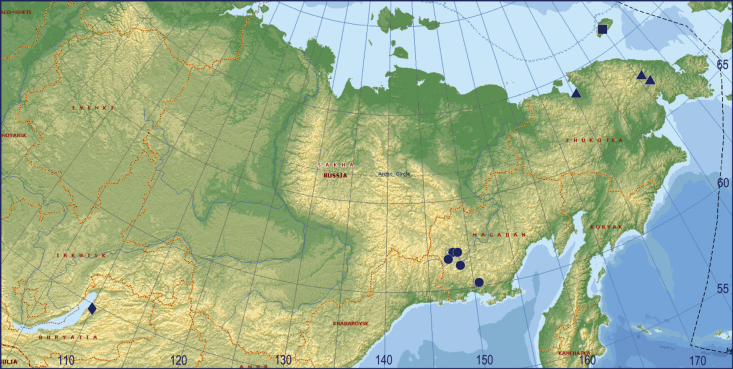
Collecting localities of *Dactylopisthoides* species: *D.hyperboreus* (dot), *D.transbaicalicus* (diamond), *D.wrangelianus* (square), records of *D.hyperboreus* that could also refer to *D.wrangelianus* (triangle).

From north-eastern Transbaikalia to Wrangel Island (Fig. 4). Its occurrence in Yakutia and Kamchatka is very likely.

#### ﻿ Dactylopisthoides hyperboreus

Taxon classificationAnimaliaAraneaeLinyphiidae

Eskov, 1990

52D0EB13-7A31-5C7A-804F-06760F7F9D83

Figs 1A–F , 3A , 4

Dactylopisthoides hyperboreus Eskov, 1990: 4, figs 1–6 (♂♀).

##### Types.

***Holotype*** ♂ (ZMMU), Russia, Magadan Area, the upper reaches of Kolyma River, Sibit-Tyellakh, *Pinuspumila* thickets, 14.IX.1985, leg. Y.M. Marusik. ***Paratypes*** (all in ZMMU): 2 ♀♀, collected together with the holotype; 4 ♂♂, 8 ♀♀, same habitat, 12.VII. 1985, leg. Y.M. Marusik; 1 ♂, upper reaches of Kolyma River, c. 10 km upstream of Vetrenny Vil., *Salix* thicket on *Carex* swamp, 5.VIII.1984, leg. K.Y. Eskov; 1 ♂, headwater of Kolyma River, Kulu River, mouth of Stokovy Creek, *Pinuspumila* thicket on scree, 11.VIII.1986, leg. Y.M. Marusik; 1 ♂, Detrin River (the right tributary of Kolyma River), c. 56 km upstream of the mouth, 30.VIII.1986, leg. Y.M. Marusik.

##### Comments.

This species has been known from a single taxonomic entry.

##### Diagnosis.

*Dactylopisthoideshyperboreus* seems particularly similar to *D.wrangelianus*. The male differs in the shorter dorsal tibial outgrowth (cf. Figs 1C and 2B) and a triangular radical tooth, vs stylet-shaped (cf. Figs 1E, 2E and Fig. 2A, B); the female differs in the wider fovea of the epigyne (cf. Fig. 1F and Fig. 2F). From *D.transbaicalicus*, it can be easily distinguished by the wider and shorter dorsal tibial outgrowth (cf. Fig. 1C and figs 40, 41, 43, 44 in Marusik et al. [2001]), as well as by the well-developed radical tooth of the embolic division (cf. Fig. 1E and Fig. 3C).

##### Description.

See Eskov (1990).

##### Distribution.

This species was described from several localities in the upper reaches of Kolyma River and Wrangel Island (Eskov 1990). Later, it was reported from Chukotka Peninsula (Marusik 1993), Ola Plateau in the south part of Magadan Area (Marusik 2005). The records of *D.hyperboreus* from Chukotka Peninsula (Marusik 1993) are likely to be erroneous and may refer to *D.wrangelianus*.

#### ﻿ Dactylopisthoides transbaicalicus

Taxon classificationAnimaliaAraneaeLinyphiidae

(Marusik, Koponen & Danilov, 2001) comb. nov.

9D10354A-4CF0-535D-A254-8280FFD2CAF8

Figs 3C , 4

Uusitaloia transbaicalica Marusik, Koponen & Danilov, 2001: 90, figs 39–48 (♂).
U. transbaicalica : Marusik and Koponen (2009): 21, figs 1c–e, 2a–c (♂).

##### Types.

***Holotype*** ♂ (ZMUT), Russia, Buryatia, Barguzin Mt. Range, Olso River, 54°52'N, 110°55'E, 1650 m, 04.VII.1996, leg. M. Uusitalo.

##### Comments.

No SEM figures are provided for this species, as it remains known from the holotype male only.

##### Diagnosis.

From *D.hyperboreus* and *D.wrangelianus*, this species differs in having the longer and narrower dorsal tibial outgrowth (cf. figs 40, 41, 43, 44 in Marusik et al. [2001] and Figs 1C, 2B) and the reduced radical tooth of the embolic division (cf. Fig. 3C and Fig. 3A, B).

##### Distribution.

Only the type locality, Transbaikalia, Russia.

#### ﻿ Dactylopisthoides wrangelianus

Taxon classificationAnimaliaAraneaeLinyphiidae

(Marusik & Koponen, 2009) comb. nov.

6F20281C-8A68-5B66-98A8-84CF5A163653

Figs 2A–F , 3B , 4

Uusitaloia wrangeliana Marusik & Koponen, 2009: 18, figs 1a, b, 2d, e (♂).

##### Types.

***Holotype*** ♂ (ZMMU), Russia, Wrangel Isl., the upper reaches of Neizvestnaya River, 71°12.933'N, 179°19.353'W, 128 m, steppe-like mound #2, 06.VII–03.VIII.2006, leg. O.A. Khruleva. ***Paratypes***: 1♂ (ZMMU), same locality, steppe-like, mound #3, 03.VII.–03.VIII.2006, leg. O.A. Khruleva; 2 ♂ (ZMMU, paratypes of *D.hyperboreus*) Wrangel Isl., lower reaches of Gusinaya River, gravel hilltop, 2.VII. 1984, leg. O.A. Khruleva; 1♂, 1♀ (ZMMU), middle reaches of Mamontovaya River, 22.VII–5.VIII.2015, leg. O.A. Khruleva.

##### Note.

Since a single female of this species was used to obtain SEM photographs, its technical description was impossible to produce.

##### Diagnosis.

*Dactylopisthoideswrangelianus* is most similar to *D.hyperboreus* (see above for diagnostic characters). From *D.transbaicalicus*, it differs in having a wider and shorter dorsal tibial outgrowth (cf. Fig. 2B and figs 40, 41, 43, 44 in Marusik et al. [2001]) and a well-developed radical tooth of the embolic division (cf. Fig. 3B, C).

##### Description.

See Marusik and Koponen (2009).

##### Distribution.

To date, this species is known from Wrangel Island only (Khruleva et al. 2022). Yet, the records of *D.hyperboreus* from Chukotka Peninsula (Marusik 1993) are likely to also refer to *D.wrangelianus*. There are also unpublished data (YM, personal data) about its occurrence in Chaun Bay in Chukotka. Unfortunately, the sample studied, represented by females, was lost.

## Supplementary Material

XML Treatment for Dactylopisthoides

XML Treatment for Dactylopisthoides hyperboreus

XML Treatment for Dactylopisthoides transbaicalicus

XML Treatment for Dactylopisthoides wrangelianus

## ﻿Additional information

### Author ORCIDs

Andrei Tanasevitch https://orcid.org/0000-0002-9116-606X

Yuri Marusik https://orcid.org/0000-0002-4499-5148

### Data availability

All of the data that support the findings of this study are available in the main text.
